# Formulation and Characterization of New Experimental Dental Composites with Zirconium Filling in Different Forms

**DOI:** 10.3390/ma17112711

**Published:** 2024-06-03

**Authors:** Dipa Rani Mohajon, Doina Prodan, Marioara Moldovan, Ioan Petean, Stanca Cuc, Miuta Filip, Rahela Carpa, Georgiana Florentina Gheorghe, Codruţa Liana Saroşi

**Affiliations:** 1 Faculty of Chemistry and Chemical Engineering, Babeș-Bolyai University, 11 Arany János Str., 400028 Cluj-Napoca, Romania; deepamj95@gmail.com (D.R.M.); ioan.petean@ubbcluj.ro (I.P.); 2 Raluca Ripan Institute for Research in Chemistry, Babeș-Bolyai University, 30 Fantanele Str., 400294 Cluj-Napoca, Romania; marioara.moldovan@ubbcluj.ro (M.M.); miuta.filip@ubbcluj.ro (M.F.); liana.sarosi@ubbcluj.ro (C.L.S.); 3 Department of Molecular Biology and Bio-Technology, Faculty of Biology and Geology, Babeș-Bolyai University, 1 M. Kogalniceanu Street, 400084 Cluj-Napoca, Romania; rahela.carpa@ubbcluj.ro; 4 Faculty of Dental Medicine, Carol Davila University of Medicine and Pharmacy, 17-23 Calea Plevnei, 010232 Bucharest, Romania; georgiana.gheorghe@umfcd.ro

**Keywords:** composites, glass fibers, zirconium, degree of conversion, AFM, SEM, antimicrobial

## Abstract

Short glass fibers are generally used in posterior dental restorations to enhance the mechanical properties and improve the material microstructure. Two resin-based composites (S0 and SF) were formulated and characterized to investigate the influence of zirconium in their characteristics and properties. The organic part of the investigated materials was the same (BisGMA, TEGDMA, and a photochemical polymerization system), and in the inorganic part, besides quart, glassA, and hydroxylapatite with Zn, sample S0 contained strontium glass with zirconium and sample SF contained fiber powder of chopped zirconium. The samples were characterized by the degree of conversion (DC), mechanical properties, water sorption (WS), scanning electron microscopy (SEM), atomic force microscopy (AFM) before and after the WS test, and antimicrobial properties. The results obtained were subjected to one-way ANOVA and Tukey’s statistical tests. Both samples had a high DC. Regarding the mechanical properties, both samples were very similar, except DTS, which was higher for the composite without fibers. After 14 days, the WS value of the SF sample was lower than that of the S0 sample. Water caused significant changes in the topography of the SF sample, but thanks to its antimicrobial properties and the diffusion phenomenon, SF had a more pronounced antimicrobial effect. This study shows that the addition of appropriate amounts of Sr-Zr-glass powder gives the material in which it is added similar properties to material containing chopped zirconium glass fiber powder. According to the antimicrobial test results, resin composites containing experimental zirconia fillings can be considered in future in vitro clinical studies for posterior reconstructions with significantly improved mechanical properties.

## 1. Introduction

Composites reinforced with short glass fibers are generally used in posterior dental restorations, having a beneficial influence by taking over the stresses that favor the polymerization contraction of the composite resin and, respectively, marginal microleakage [1]. Also, the inorganic filler (glass, quartz, hydroxyapatite, fibers, etc.) dispersed in the organic matrix has mainly a reinforcing role, obtaining superior mechanical properties [2]. In general, the content of inorganic filler in a composite can vary in the range of 50–80 wt.% and the size of the particles can have nano or micrometric dimensions.

The type of filler, the ratio between the inorganic filler and the organic matrix, and the size and shape of the particles all have major influences on the mechanical properties, radiopacity or translucency, aesthetics, antibacterial effect, etc. [3]. 

Fiber glass is also well known for its good mechanical properties [4,5]. The advantage of the resin composite is that it withstands difficult environments that differ from patient to patient with regard to occlusal habits, masticatory forces, abrasive foods, temperature changes, bacteria, and salivary enzymes, and the lifespan of a restoration depends on these factors [6]. 

There are various reasons that may affect resin composite properties, including wear resistance, microhardness, dimensional stability, color stability, and fracture resistance. Water sorption, temperature, and length of exposure to aqueous media may impact resin composite properties. The improvement in the mechanical properties of composites with fibers is due to the fact that, when the load is applied to them, the stresses that occur in the resin matrix are transferred to the fibers, which are stronger [7]. The improvement in the mechanical properties of composites with fibers is due to the fact that they are more effective crack inhibitors due to their geometry [8].

Besides the polymer initiation system, the coupling agent (silane) has a decisive role in ensuring good adhesion between the resin matrix and the fiber [9]. According to the literature data, it seems that the simple addition of fibers with micrometric dimensions to the resin matrix, together with other powders in the composition of the composite, does not improve its mechanical properties [10]. Composites like Alert and Nulite F offer a rough surface that can lead to progressive wear through fiber exposure and uneven fiber distribution. Other material, such as Short GFRC (everX Posterior, GC, Tokyo, Japan), can be considered as a dentin substitute in large cavities under the conventional composite to avoid possible fracture. It contains a matrix of resin, barium glass, and a small percentage (8.6 wt.%) of randomly oriented short fibers, showing good bending strength and fracture toughness [9]. With the help of glass fibers, the aesthetic properties and resistance of materials can be improved, as well as their biocompatibility and adaptability. 

Another study showed that by using a filler based on E-glass fibers and nano hydroxyapatite, with the increase in the proportion of added fibers, the bending strength and water absorption of the composites were negatively affected [11]. The limitations of using short fibers as reinforcing agents include compromised aesthetics because the materials cannot be polished to a high gloss, and some restorative materials can be used as dentin restorative materials [12].

Literature data show that the antimicrobial effect of restorative dental composites can be obtained in two ways: following interaction with the outer membrane of bacteria causing its damage or by rejecting bacterial adhesion or following disruption of bacterial proteins/bacterial DNA synthesis. The release of antimicrobial agents (Ag, Zn), however, can degrade the mechanical properties of the composites through the appearance of nanopores that can lead to cracks and delamination on the surface of the material, sometimes changing the shade of the restorations [13]. Time-controlled release of antibacterial agents from dental composites is important, and the addition of strontium to the powders of composite materials has been shown to have a durable effect of bacterial resistance in vitro while maintaining some of the mechanical properties of ordinary acrylic resins [14].

The novelty of the study consists of the fact that two resin-based composites with similar composition were formulated and characterized, modifying only one of the components. One composite contains strontium and zirconium glass powder, and the other composite contains chopped zirconia glass fiber powder. Following the investigation of the two materials, it was determined how glass powder with zirconium or chopped glass fiber powder with zirconium influences the properties of the two materials. We investigated the contraction, flexural, compression, and strength behaviors, water absorption, surface area, and antimicrobial effect to test the null hypothesis that the addition of chopped zirconium fiber powder will improve the characteristics of dental composite resins.

## 2. Materials and Methods

### 2.1. Materials

Two composite pastes were obtained, having the compositions indicated in Table 1.

The Sr-Zr-glass powder and the chopped Zr fiber powder were previously silanized with 3-methacryloyloxypropyl-1-trimethoxysilane (A-174 silane) from Sigma-Aldrich Chemical Co., St. Louis, MO, USA. The bioglass silanization was carried out in a porcelain mill, by pouring the silanol solution over the prepared powder and introducing it into the mill, followed by grinding for 60 min. The content obtained was dried in an oven at 30 °C, after which the fixing of silane was established in an enameled tray after 7 h of rest, followed by thermal treatment in an oven at 50 °C. The obtained material was sieved manually on a sieve with 10,000 mesh/cm^2^.

The composite material was obtained by mixing the organic and inorganic parts, and after the introduction of the photochemical system for initiating the polymerization reaction, the materials were deposited and stored in dark containers. The material transformed from a fluid liquid state to a solid state through the polymerization process in the presence of a dental LED light curing lamp (Woodpecker Medical Instrument Co., Guilin, China) for 20 s with maximum intensity at 455 nm, light irradiance 1200 mW/cm^2^.

### 2.2. Characterization

#### 2.2.1. Degree of Conversion (DC)

The determination of the degree of conversion of the organic matrix (L) and of samples S0 and SF (without and with fibers) was carried out immediately (approx. 2 min) and 24 h after polymerization. The amount of unreacted methacrylic groups was determined as a percentage of the amount of original methacrylic group present in the unpolymerized material.

The ATR-FTIR spectra of composites (pastes and solids) were recorded on an FTIR spectrophotometer (Jasco FTIR-610, Jeddah, Saudi Arabia) equipped with an ATR (attenuated total reflectance) attachment with a horizontal ZnSe crystal (Jasco PRO400S). 

Unreacted methacrylic groups were determined as a percentage of the original methacrylic groups present in the uncured material. For the quantitative determination of the unreacted methacrylate groups, the absorption band from 1635 to 1640 cm^−1^, corresponding to the valence vibrations of the C=C double bonds in the methacrylic groups (Ameth), were used. The absorption band of the phenyl group (Aarom) at 1605–1610 cm^−1^ was used as an internal standard.

The residual double bonds were calculated using Formula (1):

conv% = {1 − [Amet/Aarom]copolimer/[Amet/Aarom]monomer} × 100(1)

where A represents the absorbance intensity for: Amet—C=C double bonds in the monomer, respectively, polymer; Aarom—the C-C bonds in the aromatic ring.

#### 2.2.2. Mechanical Resistance Tests

The formulated materials were mechanically investigated using a Lloyd LR5k Plus mechanical testing machine (Ametek/Lloyd Instruments, Lübeck, Germany), which has a maximum load of 5 kN.

Three types of mechanical tests were investigated: compression resistance, resistance to diametrical compression, and flexural strength. For each test, the materials were cured under the UV lamp in different Teflon matrices to obtain specimens of different sizes, according to Table 2. A total of 10 samples were prepared for each recipe tested and each type of mechanical test. The polymerized samples were placed in tubes with artificial saliva at 37 °C for 24 h before measuring the mechanical strength.

For each group of specimens, the results were subjected to statistical analysis for the calculation of median and standard deviations. All datasets were analyzed using the one-way ANOVA test (α = 0.05), performed with Origin2019b Graphing & Analysis software (OriginLab, Northampton, MA, USA).

#### 2.2.3. Water Sorption (WS)

Experimental dental composite specimens are prepared according to ISO 4049 [16], in the form of disks with specified dimensions of 14 mm diameter and 1 mm height. Each sample was subjected to the photopolymerization process with the help of an LED.E (Guilin Woodpecker Medical Instruments Co., Guilin, China) lamp in 5 points of 20 s each. For each investigated group, 10 discs of composite material were photopolymerized.

Before testing, the samples were held in a controlled environment to achieve a stable mass. This involved storing the samples in a desiccator to remove any moisture and achieve a constant basis weight (*m*_0_). The included specimens were then immersed, individually, in 20 mL of distilled water at a specified temperature (37 °C) for 14 days.

After the immersion period (1, 2, 3, 7, and 14 days), the specimens were removed from the water, dried to remove any surface moisture, and weighed to determine their wet weight (*m*_1_). The specimens were then reconditioned in a desiccator to remove any absorbed water and obtain a stable mass (*m*_2_).

The water absorption of the dental composites was calculated using the following formula:

(2) $$Sp={(m}_{1}-m_{2})/V$$

where *m*_1_ = the mass of the sample 20 s after removal from the container; *m*_2_ = the final mass after the sample was dried in the desiccator; and *V* = the volume of the samples. 

For each group of specimens, the results were subjected to statistical analysis for the calculation of median and standard deviations. All datasets were analyzed using the one-way ANOVA test (α = 0.05), performed with the Origin2019b Graphing & Analysis software (OriginLab, Northampton, MA, USA).

#### 2.2.4. Scanning Electron Microscopy (SEM)

The perfectly dried specimens used for liquid absorption tests were used for the scanning electron microscopy investigation. All of these samples were investigated before and after storage in distilled water after a 14-day period. SEM images were obtained on the dried sample discs using an SEM-Inspect S (FEI Company, Hillsboro, OR, USA) at a magnification of ×1000, ×2000, and ×5000. The secondary electron images were obtained on uncoated samples using an acceleration voltage of 20 kV, employing a large-field detector (LFD) and low-vacuum chamber.

#### 2.2.5. Atomic Force Microscopy (AFM)

The atomic force microscopy (AFM) investigation was effectuated using a Scanning Probe Microscope JSPM 4210 produced by JEOL Co. (Tokyo, Japan). The composite disc surface was probed with the NSC 15 cantilevers produced by MikroMasch Co. (Tallinn, Estonia), having a resonant frequency of 325 kHz and a force constant of 40 N/m. The scanning was operated in tapping mode (intermittent contact mode) at a rate of 1–3 Hz depending on the scan size and sample corrugation. 

The topographic images were processed with WinSPM 2.0 processing software produced by JEOL Co. (Tokyo, Japan), and surface roughness parameters Ra and Rq [18,19] were measured for the sample’s fine microstructure (scanning areas of 20 µm × 20 µm and 10 µm × 10 µm, respectively) and for the ultra-structural details at scan sizes of about 5 µm × 5 µm and 2.5 µm × 2.5 µm, respectively. At least three different macroscopic zones were scanned for each specimen, the roughness measurements were performed for each investigated area, the mean values were calculated, and the standard deviation is represented as error bars in the variation plot along with the statistical analysis results.

#### 2.2.6. Antimicrobial Activity

Tested microorganisms. The antimicrobial activities of the S0 and SF samples were evaluated against *Staphylococcus aureus* ATCC 25923, *Enterococcus faecalis* ATCC 29212, *Streptococcus mutans* ATCC 25175, *Porphyromonas gingivalis* ATCC 33277, and *Escherichia coli* ATCC 25922 from the collection of the Microbiology Laboratory, Faculty of Biology and Geology, UBB, Cluj-Napoca.

Each bacterial strain was cultivated on nutrient agar medium at 37 °C for 24 h [20]. Then, a dilution adjusted to the turbidity equivalent to 0.5 McFarland Standard (approx. 1.5 × 10^8^ CFU/mL) was made from each strain in sterile physiological serum, according to EUCAST protocols [21].

The agar diffusion test was conducted on Müeller Hinton agar medium (from Oxoid, specific for antimicrobial testing). From the adjusted dilutions, each Petri dish was inoculated with a sterile swab soaked in the 0.5 McFarland microbial suspension, spreading over the entire surface of the solid culture medium 

**Figure 1:**
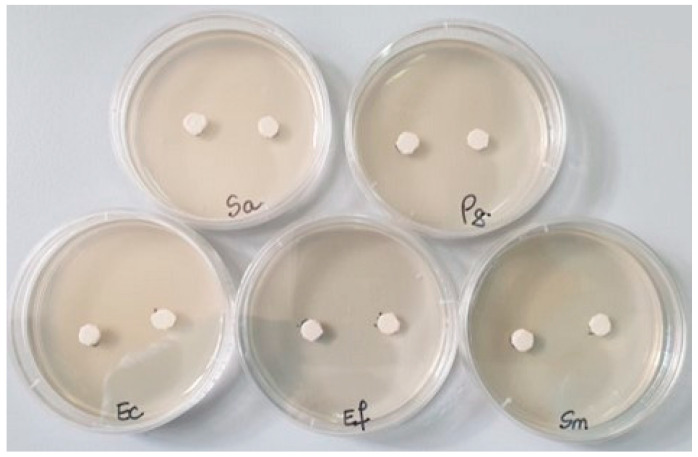
The bacterial strains studied and the samples (S0 and SF) tested (Sa = *Staphylococcus aureus*, Pg = *Porphyromonas gingivalis*, Ec = *Escherichia coli*, Ef = *Enterococcus faecalis*, Sm = *Streptococcus mutans*).

Then, in each Petri dish with the culture medium inoculated with the bacteria to be tested, the two samples (noted 1 = S0 and 2 = SF) were applied (Figure 1). Incubation was performed for 48 h at 25 °C. The reading was conducted by measuring the diameter of the inhibition zone with a millimeter ruler with an accuracy of 0.5 mm; the larger the diameter of the inhibition zone, the greater the sensitivity of the bacteria to the respective samples [22].

## 3. Results

### 3.1. Degree of Conversion (DC)

**Figure 2:**
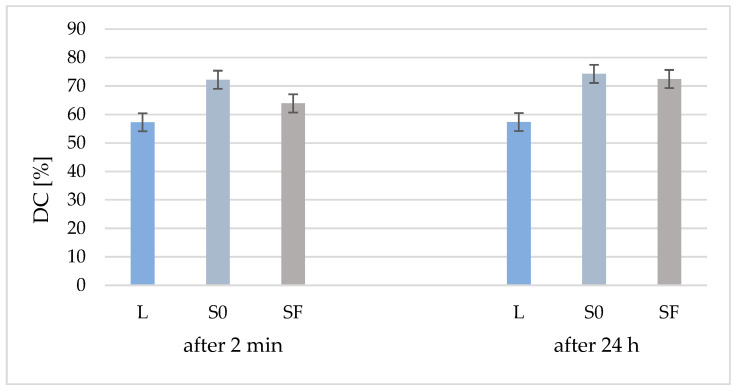
Conversion degree of organic matrix (L) and samples S0 and Sf (without/with glass fibers) at 2 min after curing and after 24 h.

From Figure 2, it can be seen that the degree of conversion (DC) of the two composites (S0 and SF) is higher than that of the polymer matrix due to the addition of the inorganic filler, both at 2 min after polymerization and after 24 h. Another observation is that, although 24 h after polymerization the DC of the polymer matrix is almost identical (57%), to that of the S0 (without fibers) and SF (with fibers) composites, the DC is increased by 2% in the case of the S0 composite and by 9% in the case of SF composite. Although the DC of the composite without fibers is higher than that of the composite with fibers after 24 h, the values are much closer (74% and 72%, respectively), which means that polymerization continues over time.

Following the statistical test for DC after 2 min, there are differences between all three analyzed samples (*p* < 0.05) and, among the results after 24 h, there are differences between the liquid and S0 and SF, but there are no differences between S0 and SF. Comparing the results according to the analysis time, there are no DC differences between the immediately hardened liquid and 24 h after hardening. The same result is obtained for the S0 sample, while for the SF sample, the DC value increases with significant differences in 24 h.

### 3.2. Mechanical Properties and Statistical Analyses

From Table 3, it can be seen that the average values of flexural strength (83 MPa) and compression strength (222 MPa) are almost identical for both series of composites (without/with fiber addition). The average value of the diametrical compression strength is higher for the series of composites with fiber-free filling (172 MPa) compared to the composites with fibers (128 MPa). The value of the modulus of elasticity is higher for the composite with fibers (SF) by 3.1 GPa compared to the composite without fibers (S0).

Comparing the statistical results, for Young’s modulus and diametrical compression resistance show significant differences between the two samples, and the values for compression resistance and bending resistance do not show significant differences.

### 3.3. Water Sorption (Sp)

**Figure 3:**
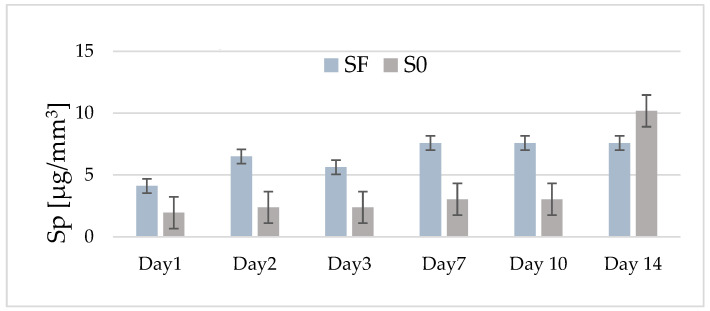
The distribution by day of the average values of water absorption for the experimental composites with fibers (SF) and without fibers (S0) in the range of 1–14 days of storage in distilled water.

Figure 3 shows the variation in the average values of water sorption for the two experimental composites (with and without fibers) depending on their storage period in water. In the case of fiber composite (SF), an increase in water sorption values is observed until the second day (6.5 μg/mm^3^), after which, on the third day, a slight decrease is observed (5.6 μg/mm^3^).

After 7 days, the average absorption value increases slightly (7.6 μg/mm^3^), remaining constant for the rest of the investigation period. In the case of the composite without fiber (S0), the average values of absorption, recorded in the interval of 1–10 days, are lower (1.9–3.03 μg/mm^3^) than those recorded in the case of the SF composite. However, it can be observed that, after this storage interval, the average values of absorption increase quite a lot, so that after 14 days, an average of 10.18 μg/mm^3^ is recorded. All of the recorded values are below the maximum limit imposed by ISO 4049/2000 [16].

The statistical tests predicted significant differences in time for both investigated samples (*p* < 0.0001). For sample S0, until day 10, there are no differences in absorption, the evolution being constant, and for sample SF, the statistics show differences between days 1, 2, 3, and 7, and 10 and 14. Comparing the absorption evolution in the 14 days between the two samples, there are no significant differences between them (*p* = 0.07313).

### 3.4. Scanning Electron Microscopy (SEM)

**Figure 4:**
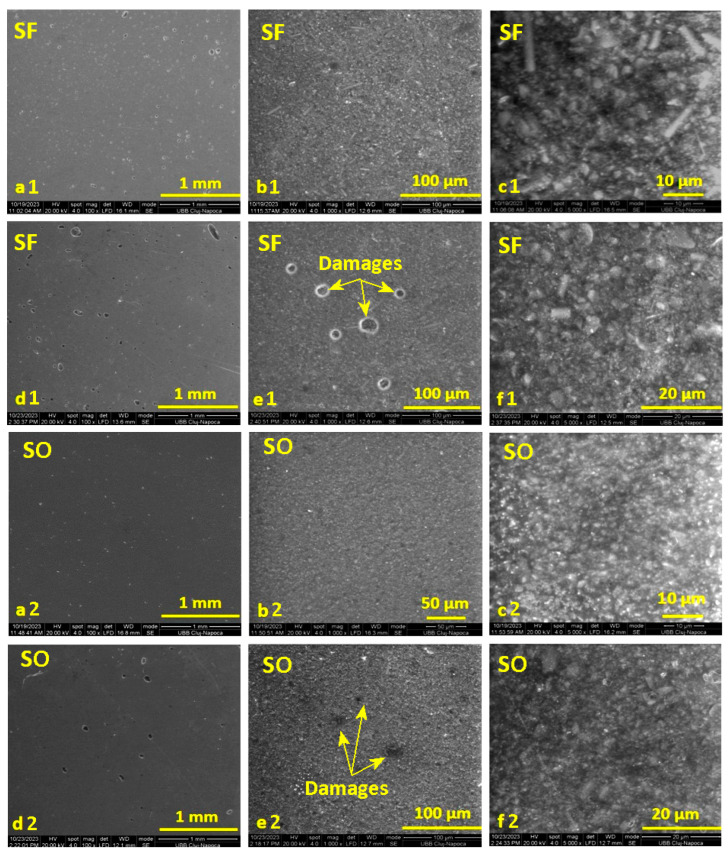
SEM images recorded on the surface of the selected samples with fibers (SF) and without fibers (S0) before immersion in water (images (**a**–**c**)) and after 14 days of storage in distilled water (images (**d**–**f**)), at magnifications of: ×100, ×1000, and ×5000.

From Figure 4, in the case of the initial images before the period of storage in water for the SF and S0 samples (images a and b), at magnifications of ×100 and ×1000, smooth surfaces of the samples can be observed, with a uniform distribution of the inorganic filler in the organic matrix. Also, in the case of image b1, for the SF sample, the presence of glass fibers can be observed, distributed randomly but uniformly in the organic matrix. Small air bubbles are also evident, incorporated during the mixing of the paste to make the samples. The initial surface of sample S0 also shows some air bubbles, but they are smaller and fewer.

In the images captured on the surface of the SF sample after absorption (images d1, e1, f1), it can be seen how the water manages to penetrate the thin surface of the organic matrix that incorporates air. In the case of image c, it can be seen that the water does not penetrate deeper into the surface layer but only in the areas with bubbles. The surface of sample S0 (images d2, e2, f2) is sprinkled with small gaps after storing the sample in water; and similarly, in this case, the water does not penetrate deeper into the surface layer but only into the small bubbles on the surface.

### 3.5. Atomic Force Microscopy (AFM)

The AFM analysis complemented the data on the morphology and surface organization of the samples obtained by SEM analysis on the composites with and without fibers.

**Figure 5:**
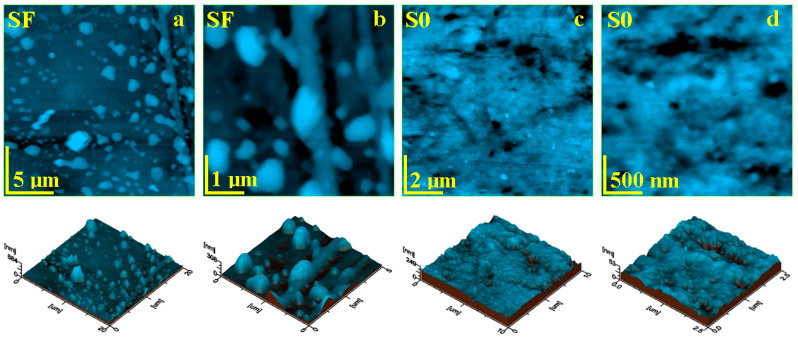
AFM topographic images obtained for the initial samples: (**a**) SF fine microstructure, (**b**) SF ultra-structure, (**c**) SO fine microstructure, and (**d**) SO ultra-structure. The three-dimensional profiles are given below each topographic image.

The topographic aspect of the initial composite samples was probed with AFM and the most relevant obtained images are presented in Figure 5. The composite with glass fibers (SF) presents a heterogeneous topography induced by the complex filler system (Figure 5a).

The highest topographical features are at the top end of the bigger glass fiber fragments, reaching local heights of about 884 nm and diameters of 3 µm. The left side of the observation field within Figure 5a reveals a smaller glass fiber having about 1 µm in diameter and length of 20 µm. These features are surrounded by micro-sized filler particles featuring a boulder shape and sizes ranging from 500 nm to 2 µm. All of these components are very well embedded into a compact mass of polymer filled with hydroxyapatite and silica nanoparticles, which are not visible at this level. The ultra-structural aspect in Figure 5b shows glass fibers embedded into the matrix surrounded by the filler particles. Both glass fiber and micro-scale filler particles are coated by a dense and consistent layer of polymer containing very well-embedded nanoparticles. The constituent’s disposal into the surface topography induces an increased roughness of Ra = 46.6 nm and Rq = 74.4 nm.

The S0 composite sample presents a relatively uniform topography (Figure 5c), formed by a compact polymeric structure that uniformly embeds the nanostructured filler system based on hydroxyapatite and silica nanoparticles. There are observed some small rounded pits over the surface caused by the sample molding, which do not penetrate into the deeper layers of the sample. Thus, the surface is very smooth, a fact sustained by a low roughness values of Ra = 18.8 nm and Rq = 24.3 nm. The ultra-structural level of the S0 sample (Figure 5d) reveals its nonstructural character. There appears to be a mixture of rounded nanoparticles of about 40 nm for silica and 60 nm for hydroxyapatite. These are very well coated by the polymer layer into a dense structure. However, there are some free exposed filler nanoparticles within the outermost layer of the sample.

**Figure 6:**
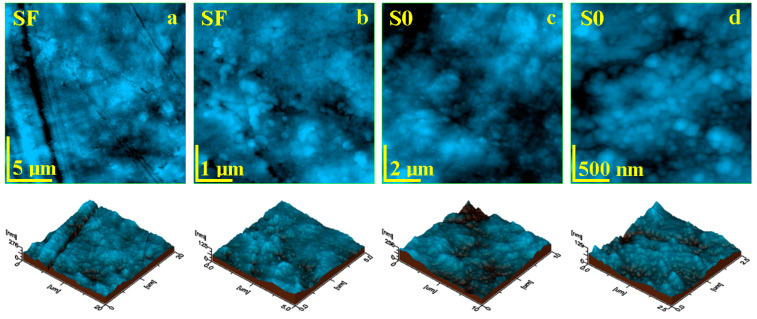
AFM topographic images obtained for the samples after 14 days of exposure to liquid: (**a**) SF fine microstructure, (**b**) SF ultra-structure, (**c**) SO fine microstructure, and (**d**) SO ultra-structure. The three-dimensional profiles are given below each topographic image.

Liquid exposure of the SF sample for 14 days causes significant changes within the sample topography. The SEM investigation revealed local delamination of the exposed filler particles as well as glass fibers within the outer most layer of the sample (Figure 4d–f). The AFM investigation revealed a significant mineral loss from the sample surface due to the complete delamination of the filler particles and their complete removal (Figure 6a). Consequentially, some depressions are observed having the shape and size of the former filler boulders, which are displaced. Such behavior would lead to a significant increase in roughness, but the depressions are filled with nanostructural debris resulted from the delamination of the nanostructural filler particles, causing a factual reduction in the surface roughness, resulting in values such as Ra = 22.3 nm and Rq = 29.6 nm.

The ultra-structural aspect of the SF sample after 14 days of wet exposure revealed that nanostructural debris is better deposited over the sample surface (Figure 6b). There are mostly nanoparticles of about 60 nm, followed by some finer ones of about 40 nm, which are related to the hydroxyapatite and silica nano-fillers. The debris has a great coalescence tendency, forming submicron clusters such as the ones observed in the left side of Figure 6b.

Open exposed nano-filler particles within the S0 sample surface are also subjected to liquid penetration between their surface and the polymer matrix, causing local delamination with subsequent mineral loss. The released nanoparticles have a great coalescence tendency and are deposited onto the sample surface as small micro clusters with the aspect of successive dunes (Figure 6c). This fact leads to a significant increase in roughness, with values such as Ra = 25.0 nm and Rq = 44.5 nm. Figure 6d reveals the topography and morphological aspect of a dune cluster, revealing its structure being composed of a mixture of nanoparticles having 40 and 60 nm diameters, corresponding to silica and hydroxyapatite nano-fillers.

**Figure 7:**
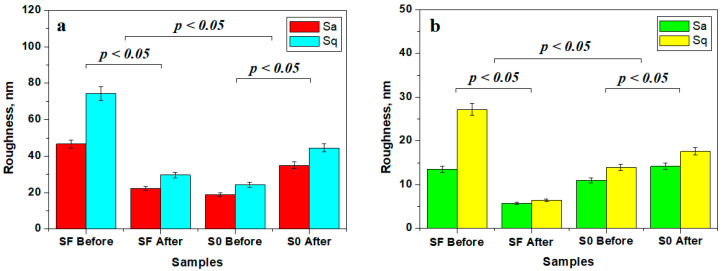
Mean roughness variation for the sample surface at the level of: (**a**) fine microstructure and (**b**) ultra-structure.

The mean roughness variation within the investigated samples is presented in Figure 7. The roughness of the fine microstructure is strongly affected by liquid exposure due to filler particle delamination leading to subsequent mineral loss. The bigger particle loss from sample FS is compensated by nano-filler particle debris deposits, causing a decrease in roughness at the fine microstructure level (Figure 7a) and at the ultra-structure level (Figure 7b). 

Conversely, S0 sample roughness is increased due to the nano-filler debris deposits, as observed in Figure 7a, but the obtained values are still low compared to the initial roughness of the FS sample. This fact indicates that the variation deals with nanostructural matter re-disposal after delamination and mineral loss. The observation is sustained by the roughness variation at the ultra-structural level (Figure 7b).

The statistical analysis of the roughness variation showed that the initial samples SF and SO form distinct statistical groups having significant differences regarding the samples after 14 days of exposure (*p* < 0.05), proving that the observed micro and nanostructural changes are significant. The statistical comparison of the SF and S0 samples showed significant differences (*p* < 0.05). Therefore, the composition of the SF and S0 samples has a great influence on their structure and behavior under liquid exposure.

### 3.6. Antimicrobial Activity

After the end of the incubation period at 25 °C, the zones of inhibition (mm) were determined for the tested microbial strains. It was observed that for all the bacterial strains studied, the tested samples show good inhibition but the inhibition zones vary in the size of the diameter depending on the tested microbial strain (Table 4).

**Figure 8:**
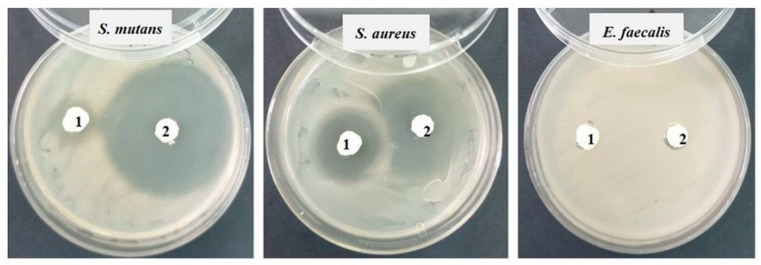
Inhibition recorded against Gram-positive strains after 48 h of incubation.

Against the Gram-positive bacterial strains *Staphylococcus aureus* ATCC 25923 and *Streptococcus mutans* ATCC 25175, fairly good inhibition is observed in both tested samples, but sample 2 (SF) shows much stronger inhibition (20–25 mm) than sample 1 (S0). Against the bacterial strain *Enterococcus faecalis* ATCC 29212, no inhibition is recorded (Figure 8).

**Figure 9:**
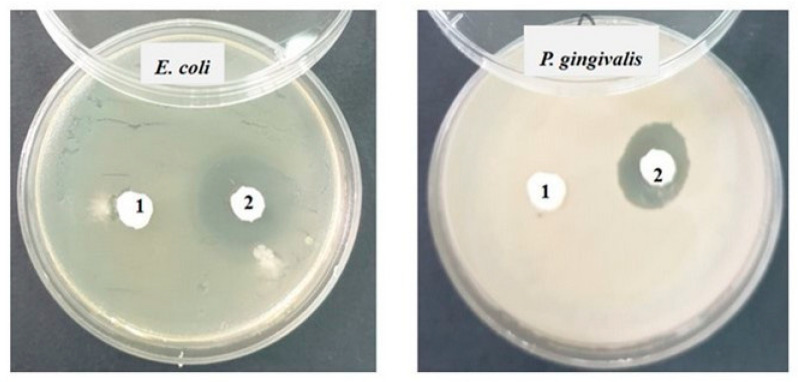
Inhibition recorded against Gram-negative strains after 48 h of incubation.

Against the Gram-negative bacterial strains *Escherichia coli* ATCC 25922 and *Porphyromonas gingivalis* ATCC 33277, the inhibition is slightly lower (12–15 mm) than that recorded against Gram-positive bacteria, and it is present only in sample 2 (SF) (Figure 9).

The tested samples show good antibacterial characteristics against the bacterial strains studied, but sample 2 has higher antibacterial activity than sample 1.

## 4. Discussion

This type of composite is recommended in posterior dental restorations, which require a reduction in the accumulated stress in that area. Therefore, a composite with crushed glass fiber filler can perform effectively in high-stress situations, providing an improvement in mechanical qualities along the convex surface. The mechanical properties of dental materials are strongly influenced by a number of factors that should be taken into account. The degree of loading, the type of filler, the method, and the time of polymerization are just some of them [23].

The size of the cut zirconium glass fibers added in the SF composite in our study is mostly about 50–100 μm in length, their orientation being random, and their distribution influenced the mechanical properties of the composite. According to the mechanical tests, it appears that the addition of glass fiber powder in the SF composite has a similar effect to the addition of Sr-Zr-based glass powder in the S0 composite. The fact that the average DTS value of the composite without fibers (S0) is 44 MPa higher than that of the composite with fibers (SF) may be due to the inhomogeneous distribution of fibers in the cured samples.

Lassila et al. have carried out investigations on some commercial composites, including SFRC, Alert, NovaPro-Flow, NovaPro-Fill, everX Flow, and everX Posterior, which contain short fibers (μm, nm, mm), obtaining high flexural strength values for composites, with the highest amounts of barium-based glass filler and micrometric glass fibers (everX Flow) [1,24]. NuliteF, a BIS-GMA-based hybrid composite loaded with E-glass fiber micro-rods with a low volume percentage of resin (29%), 35% glass filler, and 36% chopped E-glass fibers, had a high modulus of elasticity of 16 GPa [25].

Literature data show that glass fibers help to strengthen resin-based composites, but water absorption and the release of soluble oxides appear as weak points. If these aspects are not resolved, the penetration of water into the structure of the material will have the consequence of weaker mechanical properties being obtained under the conditions of the oral environment [2].

It is assumed that as the concentration of the hardening agent increases, the spaces created between them make it difficult for the organic matrix to penetrate inside them, thus favoring the penetration of water, with vulnerability in that area. Water penetrating inside these spaces helps to debond the interface between the matrix and the filler, leading to dissolution of the filler with which the water comes into contact, and then to corrosion of the material [11].

High water sorption, therefore, favors the dissolution of functional powders, but at the same time, it affects the degradation of the polymer matrix, especially if the polymerization conversion is low. Under the action of water, over time there is a danger of releasing some residual monomers that can affect the biocompatibility of the material [26].

These two factors can affect the surface hardness of the material, affecting the abrasion resistance of the material and favoring the formation of a rougher surface and bacterial adhesion that then leads to the appearance of secondary caries [27].

The topographical aspects were better observed by AFM investigation, wherein the glass fibers bonding to the polymer matrix were observed for the SF sample. The removal of filler particles led to an increase in roughness, as seen in Figure 6. This fact is in agreement with literature data [28]. Long-term liquid exposure subjects the outermost filler particles to direct contact with the liquid, which tends to infiltrate between the glass fibers and the polymer matrix, causing a significant decrease in surface roughness due to microstructural uniformity implying a smoother surface [29]. The compactness of the polymer matrix ensures liquid-resistant behavior of the unexposed filler particles and preserves the integrity of the composite sample.

On the other hand, sample S0 has a very compact and uniform surface based on a fine dispersion of mineral filler well embedded in the polymer matrix, ensuring improved compaction and smoothness, evidenced by low roughness values. Long-term liquid exposure erodes the S0 composite surface, delaminating exposed filler particles that are loosened from the structure and consequently forming debris deposits, causing a significant increase in roughness. Erosion of nano-filler as a result of exposure to liquid has been previously evaluated in the literature [30,31]. Nano erosion was preserved only on the surface of the sample, avoiding a propagation in depth due to the compactness of the polymer matrix on the constituents of the ultrastructure.

The improvement of dental composites using materials with antibacterial properties has caused rapid growth in dental studies because they can improve biological and mechanical properties [11,32,33]. Inorganic materials with antibacterial properties include metal oxides, metals, and metal phosphates [34]. Various types of metal oxide nanoparticles or metallic nanoparticles are linked for use in the fabrication of dental composites [35,36,37]. Materials developed with functionalized glass fibers, which leads to strong interfacial bonding between fibers and the resin matrix, were employed to enhance the mechanical properties of dental composites [32]. Including materials in the structure of dental composites caused an improvement in strength, resistance to body fluids, and corrosion resistance, while also providing antibacterial properties [34,38].

Substituted hydroxyapatite offers additional advantages through the presence of other elements in its structure. Zinc can easily replace Ca^2+^ ions in hydroxyapatite because it has a relatively smaller ionic radius than Ca^2+^. Therefore, HAP with Zn can be considered a bioceramic powder with antimicrobial potential capable of ensuring faster healing [39]. Some studies report antibacterial activity against *Staphylococcus aureus* (Gram positive) and *Esherichia coli* (Gram negative). The diameters of the bacterial inhibition zones vary in the first case between 11 and 14 mm and in the second case between 21 and 23 mm [40,41].

## 5. Conclusions

This study compared the influences of chopped Zr glass fibers versus Zr-Sr-based glass on the structure and properties of a composite material. Initially, the sample containing chopped Zr glass fibers (SF sample) had a lower DC than the sample with Zr-Sr-based glass (S0 sample). However, after 24 h, the DC of the SF sample increased significantly, nearly matching that of the S0 sample. The flexural strength (FS), compressive strength (CS), and Young’s moduli of both samples were found to be similar, indicating that the addition of chopped Zr glass fibers did not significantly alter these properties.

After 14 days, the water sorption of the SF sample was lower than that of the S0 sample, indicating potentially better resistance to water ingress in the composite containing chopped Zr glass fibers. Both tested samples exhibited good antibacterial characteristics against the studied bacterial strains.

Despite observed changes in surface morphology and detachment of particles, the water sorption values for both samples remained comparable to literature data. The study demonstrates that the addition of chopped Zr glass fibers can significantly influence the structure and properties of the composite, providing insight for potential applications and optimization in composite material engineering. The Sr-Zr-glass powder added to the material resulted in similar properties as the material containing chopped zirconium glass fiber powder. This suggests that both additives can impart comparable structural and mechanical properties to the composite material.

## Figures and Tables

**Table 1:** The compositions of the investigated composite materials.

Recipe	Organic Phase (25%)	Inorganic Phase (75%)		Initiation System (% Recipe)
S0	Bis-GMA 65%; TEGDMA 35%.	Glass A 20%, Quartz 40%, HA-Zn 10%.	Sr-Zr-glass powder 30% (particle size 0.01–0.035 µm and 2–6 nm)	1% DMAEM 0.5% CQ
SF			Chopped Zr fiber powder 30% (50–100 µm length)	

Bis-GMA (bisphenol A-glycidyl methacrylate), synthesized in the Polymeric Composites Laboratory of UBB-ICCRR (mixture of higher oligomers: 81% monomers, 17% dimers, and 1% trimers); TEGDMA (triethylene glycol dimethacrylate), Sigma-Aldrich, St. Louis, MO, USA; Glass A (42% SiO_2_, 26% CaO, 8% P_2_O_5_, and 24% Na_2_O), synthesized in the Polymeric Composites Laboratory of UBB-ICCRR; Quartz, Chimexin; HA-Zn (hydroxyapatite with zinc), synthesized in the Polymeric Composites Laboratory of UBB-ICCRR; Sr-Zr-glass powder (strontium-zirconium glass powder), synthesized in the Polymeric Composites Laboratory of UBB-ICCRR [15]; Chopped Zr fiber powder (chopped zirconium glass fiber powder), synthesized in the Polymeric Composites Laboratory of UBB-ICCRR; DMAEM (dimethylaminoethyl methacrylate), Sigma-Aldrich; CQ (camphorquinone), Sigma-Aldrich.

**Table 2:** The mechanical tests specimens.

Test	No. of Specimen	Specimen Dimensions	Mechanical Test Characteristics
Flexural strength *	10—S0 10—SF	2 mm height, 2 mm width, 25 mm length	3-point test compression test with a speed of 0.5 mm/min
Compression strength **		6 mm height, 3 mm diameter	compression test with a speed of 1 mm/min
Diametral compression streangth **		3 mm height, 6 mm diameter	compression test with a loading rate of 0.5 mN/s

* according to ISO 4049/2000 Standard [16]. ** according to the American Dental Association (ADA) Standard No. 27 [17].

**Table 3:** Mechanical properties (flexural, compression, diametral tensile strength, and Young’s modulus) and standard deviation (SD).

Sample	FS ± SD [MPa]	Young’s Modulus ± SD [GPa]	CS ± SD [MPa]	DTS ± SD [MPa]
S0	83.00 ± 14.456	21.4 ± 3.039	222.74 ± 19.464	172.02 ± 21.242
SF	83.85 ± 15.327	24.5 ± 5.509	222.79 ± 27.721	128.03 ± 15.265
Anova *p* value	0.94	0.02	0.96	0.00009

**Table 4:** Diameters of the inhibition zones (mm) of the tested samples and standard deviation (SD).

Bacterial Strain		S0 (±SD)	SF (±SD)
	Samples		
*Staphylococcus aureus* ATCC 25923		17 (±0.2)	20 (±0.2)
*Enterococcus faecalis* ATCC 29212		0 (±0)	0 (±0)
*Escherichia coli* ATCC 25922		0 (±0)	15 (±0.2)
*Streptococcus mutans* ATCC 25175		12 (±0.1)	25 (±0.3)
*Porphyromonas gingivalis* ATCC 33277		0 (±0)	12 (±0.3)

## Data Availability

The data presented in this study are available upon request from the corresponding author.
